# Nanoclay/Polymer Composite Powders for Use in Laser Sintering Applications: Effects of Nanoclay Plasma Treatment

**DOI:** 10.1007/s11837-017-2408-5

**Published:** 2017-06-12

**Authors:** Alaa Almansoori, Candice Majewski, Cornelia Rodenburg

**Affiliations:** 1 Department of Material Science and Engineering, University of Sheffield, Sheffield, UK; 2 Southern Technical University, Basra, Iraq; 3 Department of Mechanical Engineering, University of Sheffield, Sheffield, UK

## Abstract

Plasma-etched nanoclay-reinforced Polyamide 12 (PA12) powder is prepared with its intended use in selective laser sintering (LS) applications. To replicate the LS process we present a downward heat sintering (DHS) process, carried out in a hot press, to fabricate tensile test specimens from the composite powders. The DHS parameters are optimized through hot stage microscopy, which reveal that the etched clay (EC)-based PA12 (EC/PA12) nanocomposite powder melts at a temperature 2°C higher than that of neat PA12, and 1–3°C lower than that of the nonetched clay-based nanocompsite (NEC/PA12 composite). We show that these temperature differences are critical to successful LS. The distribution of EC and NEC onto PA12 is investigated by scanning electron microscopy (SEM). SEM images show clearly that the plasma treatment prevents the micron-scale aggregation of the nanoclay, resulting in an improved elastic modulus of EC/PA12 when compared with neat PA12 and NEC/PA12. Moreover, the reduction in elongation at break for EC/PA12 is less pronounced than for NEC/PA12.

**Electronic supplementary material:**

The online version of this article (doi:10.1007/s11837-017-2408-5) contains supplementary material, which is available to authorized users.

## Introduction

Clay nanocomposites have gained much attention in recent decades. When made through melt-compounding processes, via extrusion or injection molding, enhancement of the properties of the melt-compounded objects have been reported.^1^^–^ 3 However, challenges involved in the fabrication of complex geometries have also been recorded.^4^ Compared to the conventional techniques mentioned above, laser sintering (LS) can create highly complex geometrical parts and does not require any post-machining.^5^^,^ 6 Unlike other methods, LS, as an additive manufacturing technique, uses 3D CAD from a computer connected to the machine, to form three-dimensional parts in a layer-by-layer process.^7^^,^ 8 In LS processing, laser power and powder bed temperature have to be carefully adjusted. The powder bed temperature is held below the powder melting temperature,^6^ and is used to preheat the powder, whereas the laser is used to fuse polymer particles together.^5^^,^ 6 ^,^ 9 Preheating is essential to reduce the thermal gradient between the sintered and non-sintered powder and to reduce the laser power needed to melt the powder.

Although many studies on LS have focused on thermoplastic polymers, particularly semi-crystalline thermoplastics, due to their low melting temperature such as polyamides (nylons),^7^^,^ 8 ^,^ 10 few studies have been conducted on the reinforcement of polymers with nanofillers for LS in order to improve the mechanical properties of neat polymers by creating polymer nanocomposites. Polymers have been filled with different types of nanomaterials such as carbon nanotubes^11^^,^ 12 or carbon nanofibers.^13^ Among all the nanofillers, nanoclay (mostly montmorillonite) is the most commonly used because of the remarkable changes exhibited by the polymer after adding a small amount of nanoclay.^5^^,^ 14

Montmorillonite (MMT) is an inorganic, layered silicate and the hydrophilic clay interacts only weakly with organic polymers (typically hydrophobic ones); it tends to aggregate to form large agglomerations in the matrix. Therefore, very few studies have investigated polymers filled with pristine MMT (nontreated).^2^^,^ 15 Chemical modification of the pristine MMT via surfactants is mostly used to change the hydrophilic MMT to organophilic by exchanging the interlayer cations with organic cations (different kinds of surfactants were used).^1^^,^ 2 ^,^ 16 ^,^ 17 Although surface modification of the MMT has improved the interaction between clay and polymer, chemical modification has also been reported^15^ to be expensive; hence, alternative processes are of interest.

Previously, very few attempts have been made to treat clays using a different method, i.e. plasma treatment,^18^^,^ 19 and there have been only a few attempts at using the plasma-treated clay to prepare polymer nanocomposites.^20^^,^ 21 However, none of those studies used the treated nanoclay to prepare the polymer/nanoclay nanocomposite through a LS process.

Here, we describe and employ a downward heat sintering (DHS) process using a hot press to process small quantities of dry mixed clay/Polyamide 12 (PA12) powders into tensile test specimens after optimization attempts based on differential scanning calorimetry (DSC) and hot-stage microscopy (HSM).^22^ We also demonstrate that DHS results can be successfully applied to adjust the LS bed temperature to allow the fabrication of clay/PA12 nanacomposites.

Tensile tests were used to determine the strength, elastic modulus, and elongation at break,^2^^–^ 4 and some of the published results related to the current work, in comparison with our results, are summarized in Table S1 (supporting information) and discussed in this paper.

## Materials, Preparation Methods, and Experimental Work

### Materials and Preparation Methods

An organically modified layered silicate nanoclay used in the current study is known commercially as Cloisite 30B (C30B). It was obtained from Southern Clay Products. Virgin Polyamide 12 [trade name is Nylon 12 (N12)], the matrix, was purchased from EOS (e-Manufacturing Solution). However, the polymer used in this study was not virgin, it had previously been exposed to a high temperature in a LS at least twice, but the powder was still good quality and the same batch was used for all trials to ensure consistency.

The materials (PA12 and C30B) were processed together to make nanocomposites using simple, easy and low-cost methods comprising three parts: clay treatment and modification, dry mixing and finally sample fabrication.

- Clay treatment: Plasma treatment technique

The C30B powder was treated for 30 min before being mixed with PA12 powder. Plasma treatment was carried out in a Plasma Cleaner Zepto (from Diener Electronic) with the following parameters: max power: 100 W, pressure: 0.2–0.4 mbar, time period: 1000 s for each session, and process gas: air.

(b) Mixing: Dry mixing to obtain composite powder

Etched (EC) and nonetched (NEC) C30B were added to the neat PA12 in small glass jars (50 ml) as per the concentrations (3 and 5 wt.%) shown in Table I. The composite powders were then stirred using a magnetic stirrer for 30 min at 800 rpm and sonicated for another 30 min using an ultrasonic bath. The resulting powder was stored in a sealed glass jar for <2 weeks.

**Table I:** Technical specifications of DHS

Materials	Lower plate		Upper plate		Sample per session
	Time (min)	Temp (°C)	Time (min)	Temp (°C)	
Neat polyamide 12 (PA12)	15	185	15	190	3
(3% NEC^a^/PA12)	15	188	15	192–195	3
(5% NEC/PA12)	15	188	15	192–195	3
(3% EC^b^/PA12)	15	188	15	192	3
(5% EC/PA12)	15	188	15	192	3

^a^NEC is nonetched nanoclay.

^b^EC is etched nanoclay.

(c) Sample fabrication method: Downward Heat Sintering (DHS)

The composite powders and the neat powder were formed into tensile test specimens in a hot press, which was used to mirror the laser sintering process, and therefore no additional pressure was applied during the sample fabrication. A stainless steel hollow mold was used to make tensile test samples according to the British Standard (BS ISO 527) and it was closed from one side by a removable thick plate.

Neat PA12 and composite powders (weight ratios are given in Table I), respectively, were placed in the mold and then the mold and the powder were placed in between the two parts of the hot press. The powders were preheated by the lower part only, which was at a temperature of 185°C for the neat PA12, and 188°C for PA12 composites, before the upper part (temperature is 190°C for the neat PA12 and 192–195°C for PA12 composites) was brought down. From the point at which the upper part comes into contact with the lower part, the preheated powder will be in a closed heated chamber similar to the laser sintering chamber. As a result, the powder temperature will then rise to just above the melting temperature until being fully melted, after which, the two hot press parts will release. Finally, the parts are removed from the mold using a stainless steel spatula and left to cool to room temperature. Times and temperatures of DHS are shown in Table I.

### Experimental Work

- X-ray Diffraction (XRD) and Scanning Electron Microscopy (SEM)

XRD of powder and solid samples was carried out on a Siemens D5000 (Cu, GAXRD). X-ray scans were obtained at room temperature from 2*θ* = 2°–27° in steps of 0.02^o^ with a dwell time of 1 s per step. The machine was operated at 40 kV and 40 mA. The obtained data were analyzed using the DIFFRAC.EVA application from Bruker.

Morphological investigations were conducted using a Nova NanoSEM (Low-voltageSEM)). Two different detectors were used: a through-lens detector (TLD) for secondary electron imaging at low magnification and a concentric back-scatter detector (CBS) using back-scattered electrons to obtain high-magnification images. The TLD is normally used for topography imaging whereas the CBS is for chemical analysis.^23^

(b) DSC and HSM

A DSC 8500 from Perkin Elmer and a HSM (BX50 light microscope from Olympus with temperature controlled stage from Linkam attached) were used to optimize the melting temperature of PA12 and its nanocomposites. Melting and cooling curves were collected using associated software (Pyris^TM^). Samples for both DSC and HSM were heated from ambient to 250°C with a rate of 10°C/min.

(c) FTIR and TGA

TGA and FTIR were used to investigate the effect of plasma treatment on the nanoclay decomposition process. FTIR analysis was carried out by recording 10 scans between 400 cm^−1^ and 4000 cm^−1^ using a PerkinElmer Frontier spectrophotometer. TGA was conducted by Pyris from PerkinElmer.

(d) Tensile Testing

The tensile test was carried out to evaluate the mechanical properties of DHS samples using a Hounsfield Tensometer according to BS ISO 527. The test parameters used were: load cell was 10,000 N, the speed of test was 5 mm/min and a preload 5 N.

## Results and Discussion

### Optimization of Processing Conditions by Hot Stage Microscopy

**Fig. 1:**
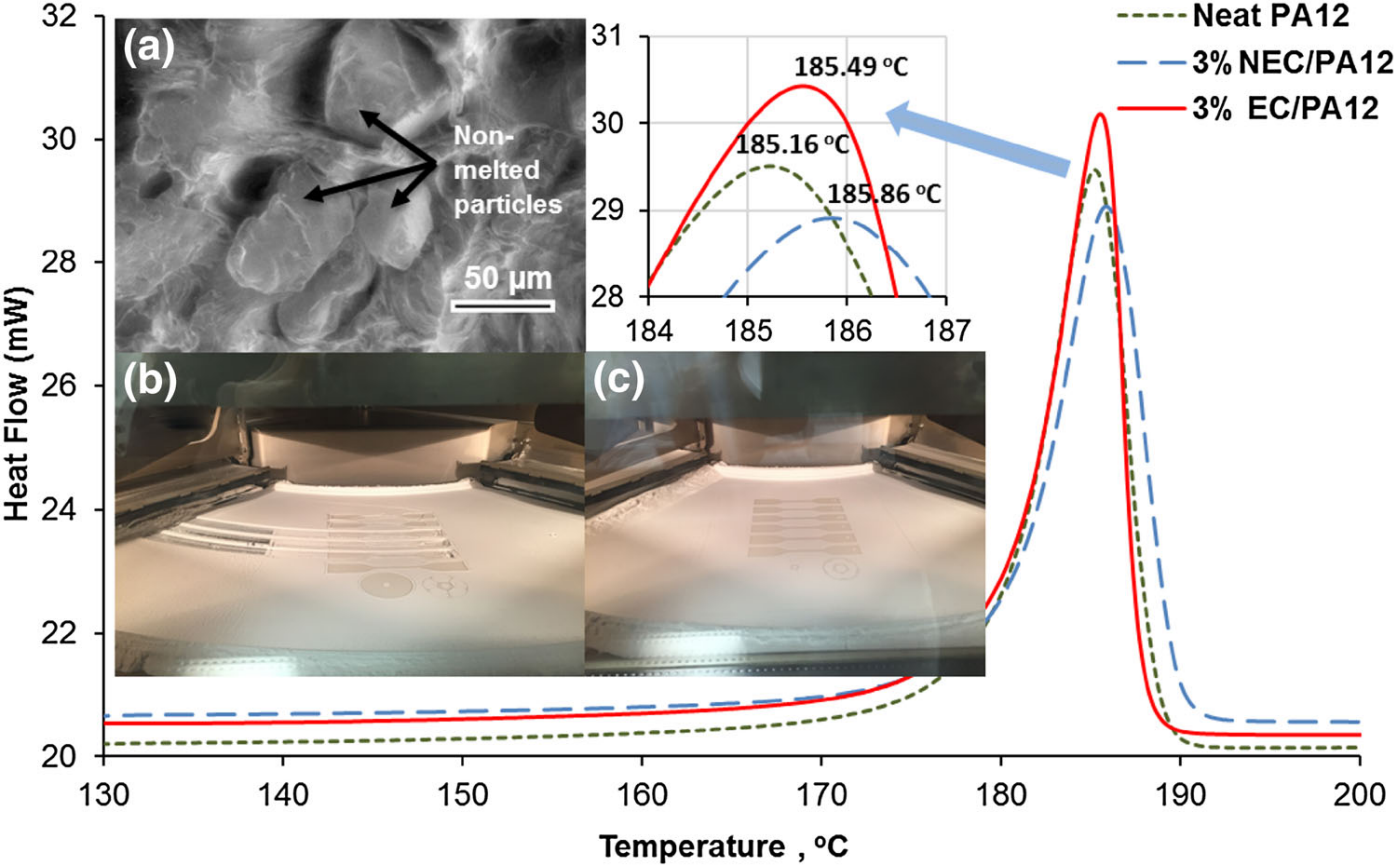
Melting temperature for PA12 and its composites measured by DSC. Inset (*a*) is a SEM image of a cross-section of a NEC/PA12 tensile test sample with non-melted particles; sample was made at a temperature suitable for neat PA12. Inset (*b*) unsuccessful LS attempt for printing NEC/PA12 at neat PA powder bed temperature. Inset (*c*) successful LS attempted for EC/PA12 composite at DHS adjusted powder bed temperature

To determine the most suitable process temperature for the fabrication of parts from the composite powder, it is necessary to use a technique that is most similar to the melting process during fabrication. Although the DSC is commonly used to quantify the melting behavior of samples in both melt processing^1^^,^ 24 ^,^ 25 and powder sintering,^5^^,^ 7 ^,^ 14 ^,^ 26 we found that the melting temperatures obtained from the DSC did not result in fully melted powders in HSM (see Fig. 1). The DSC results showed a single endotherm peak for each sample with different intensities. The average melting temperature at peaks and the onset points for all samples are almost the same, as shown in Fig. 1 (Neat PA12, 3% NEC/PA12 and 3% EC/PA12 composites).

**Fig. 2:**
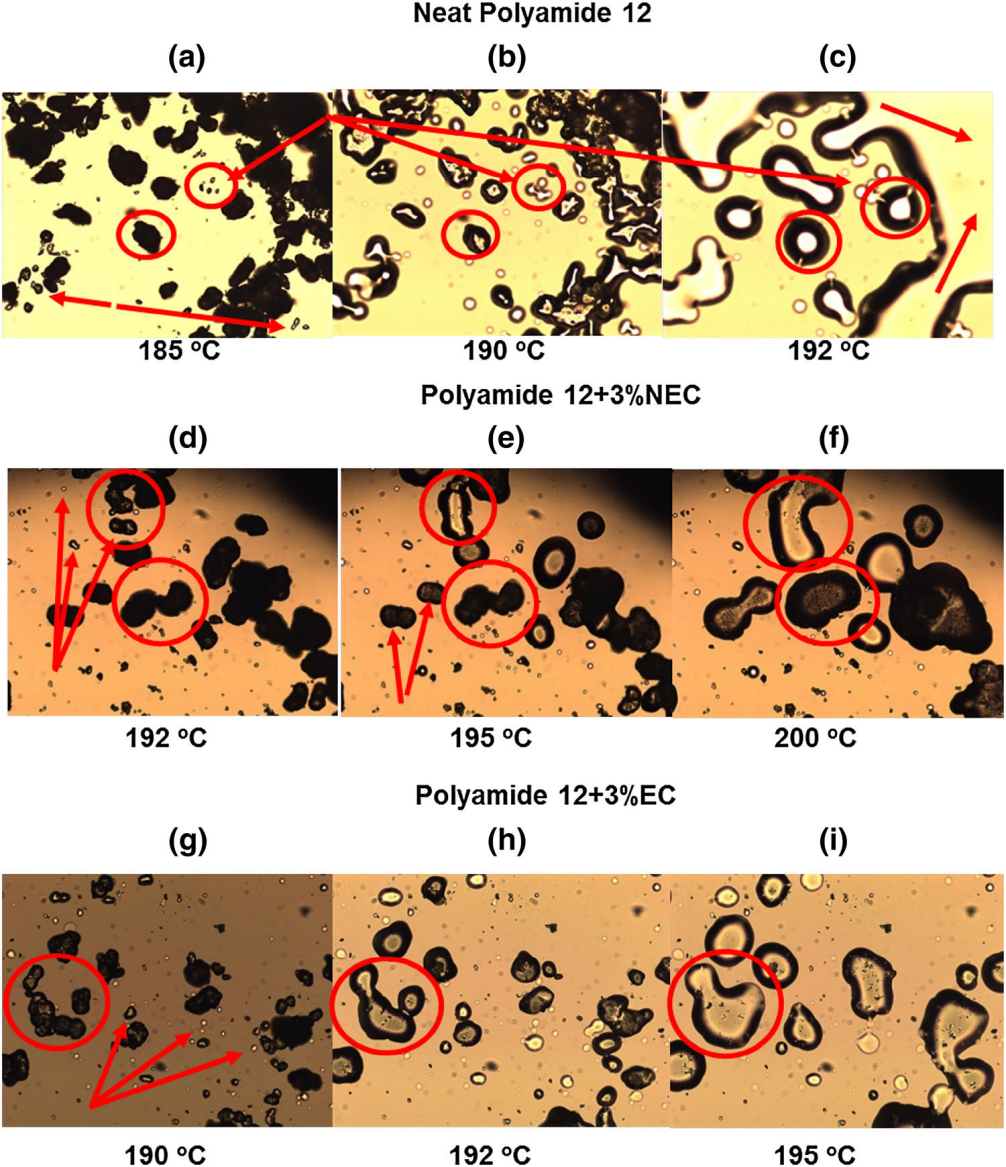
HSM results for PA12 and its composites at different temperatures. Neat PA12 at temperatures (a) 185°C, (b) 190°C, and (c) 192°C, 3% NEC/PA12 at temperatures (d) 192°C, (e) 195°C, and (f) 200°C, and 3% EC/PA12 at temperatures (g) 190°C, (h) 192°C, and (i) 195°C

The single endotherm peak corresponds to the γ crystal form.^7^ The peak positions were just above 185°C with a variation <1°C. However, the melting temperature observed during HSM was different, revealing a much larger variation between neat PA12 and the two different composite powders, as shown in Fig. 2a–i.

A clear difference was observed between the mixtures, whereby the initial and final melting temperatures increased from neat PA12 to 3%NEC/PA12 to 3%EC/PA12. For these three mixtures, respectively, melting began with micron-size particles at 185°C, 192°C and 190°C (Fig. 2a, d, and g), larger particles were partially melted and necks were formed between adjacent particles at 190°C, 195°C and 192°C (Fig. 2b, e, and h), and the melting process was completed at 192°C, 200°C and 195°C (Fig. 2c, f, and i). This is in stark contrast to the DSC results that do not show such clear differences. Compared to NEC, the EC composite powder resembles more closely the processing conditions for neat PA12, whereas the NEC composite powder required substantially higher temperatures.

As mentioned previously, the aim is to replicate the melt processing of powder in the hot press. In the HSM, the powder is heated in an open environment, similar to the initial stages of DHS, whereas DSC takes place in a fully sealed environment. That the HSM delivered more reliable input for both the DHS process and the LS is evident in Fig. 1(inset c), which shows successful LS attempted for the EC/PA12 composite when the powder bed temperature was increased by 2°C compared to neat PA12.

### Effect of Plasma Treatment on the Nanoclay (Characterization Techniques)

**Fig. 3:**
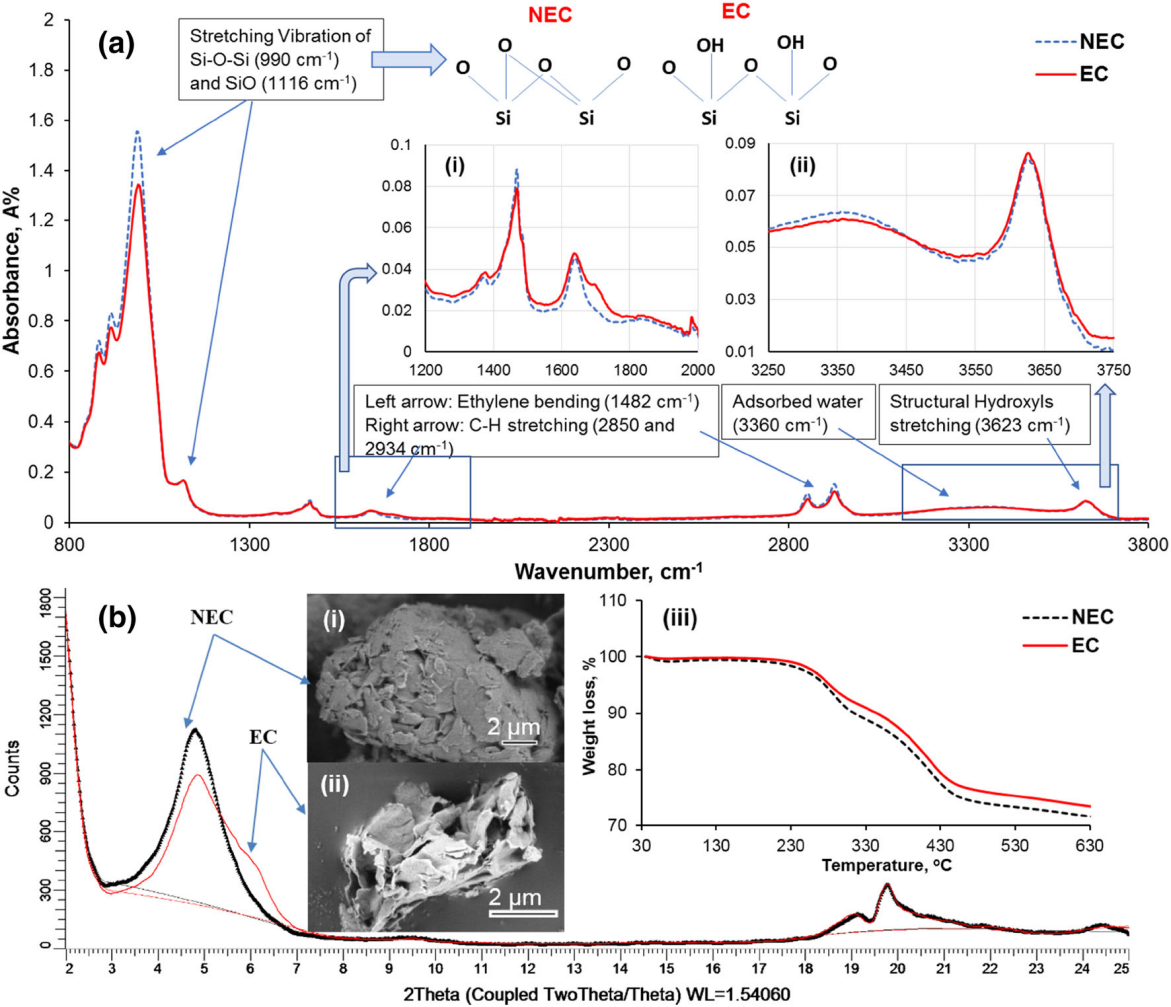
Influence of plasma treatment on the nanoclay using different characterization techniques; FTIR (a) and inset *i* and *ii* with magnified scale, XRD (b), SEM (inset *i* and *ii* in b) and TGA (inset *iii* in b)

The FTIR spectra shown in Fig. 3a indicate the presence of structural changes resulting from subjecting C30B to 30 min of plasma treatment. A significant decrease of the stretching vibration of the Si–O-Si bonds (990 cm^−1^) and some reduction in Si–O bonds (1116 cm^−1^) are observed in EC compared to NEC. These reductions suggest the introduction of lamellae disorder,^21^ which can also explain some of the observed broadness of the XRD peak of EC (Fig. 3b).^21^^,^ 27 The change in the chemical structure was not limited to the silicate band but also led to the formation of new hydroxyl groups, as evidenced by a small increase in intensity at 3623 cm^−1^ in the EC spectrum (Fig. 3a-ii). These might be the reason for the new shoulder appearing in the XRD pattern of EC in Fig. 3b. The FTIR also showed a decrease at the peak 3360 cm^−1^ that revealed a reduction in the adsorbed water. The peaks associated with the organo-modifier were also changed. The peak at 1640 cm^−1^ (the stretching of the quaternary ammonium salt) was slightly decreased, and a new peak was observed at wave angle 1695 cm^−1^. The interpretation of this change is the formation of carboxyl from the carbon of the organic modifier.^21^

Figure 3b shows the XRD diffraction patterns for both plasma-treated and non-treated nanoclays (EC and NEC). At high angles, the XRD spectra of NEC and EC exhibited two weak peaks at positions (2*θ* of 19.7° and 2*θ* of 19°). While, at low angles, the XRD patterns of NEC and EC are different, although both exhibited the same characteristic basal diffraction at 2*θ* of 4.8° and the interlayer spacing (d-spacing) of those peaks was equal to 1.8 nm (001 crystal lattice). The peaks for NEC are in good agreement with previous studies.^5^^,^ 14 ^,^ 28 The pattern of EC shows a much broader diffraction peak, consisting of a peak at 4.8° and shoulder (2*θ* of 6°). The formation of the shoulder is probably attributable to a breakdown of Si-O-Si bonds and a formation of new hydroxyls (Si-OH).^19^

Moreover, the plasma treatment can induce oxidation of the octahedral iron leading to the release of interlayer cations.^19^ The effect of the oxidation in EC can be observed by the color change of the nanoclay particles from an off-white to a gray color. The formation of the oxidative layer on the EC due to plasma treatment might lead to less absorption of moisture, which could result in an improvement of the thermal stability, which can be tested by TGA. The TGA results (Fig. 3b-iii) show that both EC and NEC are degraded in four stages, i.e. desorption of water, dehydration of hydrated cation, loss of surfactant, and dihydroxylation.^16^ For a given temperature, the weight loss of EC is always smaller than in NEC. Hence, the EC is more stable than the NEC, even at higher temperatures. In addition, TGA results corroborate our FTIR results, which indicated that plasma treatment releases some of the free water.

It is noted that the platelets of untreated clay were stuck together to form microsized agglomerates, as shown in the SEM image in Fig. 3b-i. Such agglomerations reduce the surface contact area between the clay and polymers and can weaken the composite.^5^ In contrast, the SEM image of the EC (Fig. 3b-ii) reveals the separation into platelets resulting in a much increased surface area.

### Investigation of the Properties of the Etched Nanoclay/Polymer Composites Powders

**Fig. 4:**
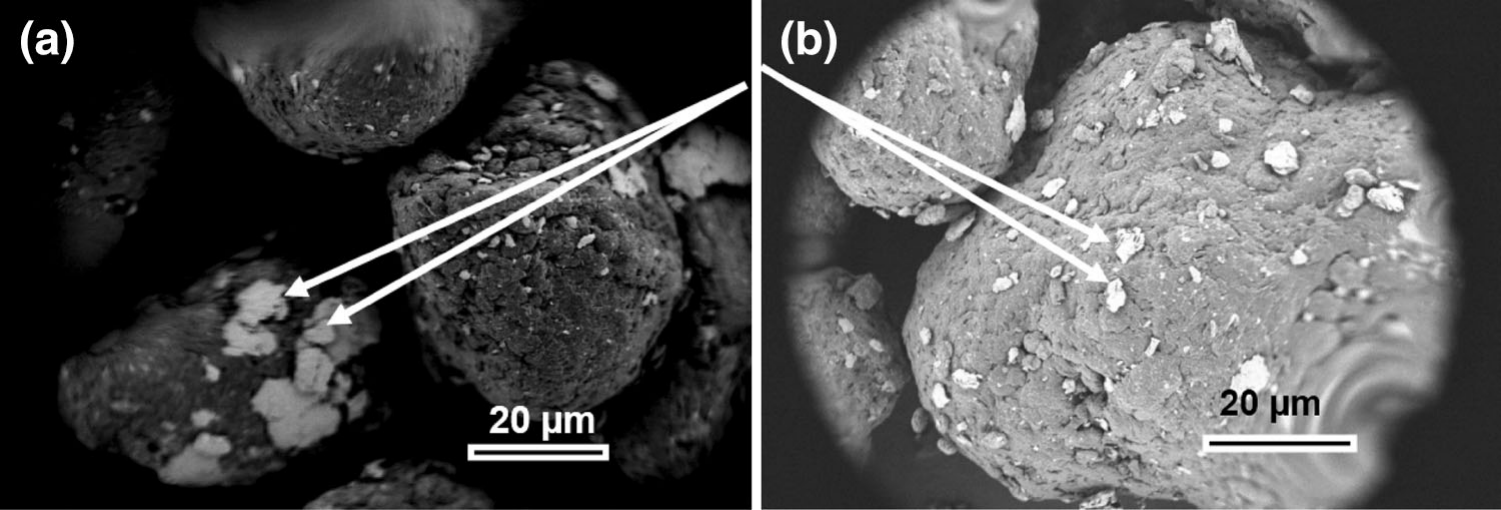
SEM images of 3%NEC/PA12 mixed powders (a) and 3%EC/PA12 mixed powders (b)

The composite powders that were made via dry mixing were investigated by XRD and SEM. The back-scattered (BSE) SEM images in Fig. 4a, and b show the incorporation of platelet-shaped nanoclay in the circular or potato-shaped PA12 particles. SEM-BSE images reflect the average atomic number. As nanoclay is largely a mineral material, whereas PA12 is an organic material, the nanoclay appears bright.

For the NEC, we find stacks of clay platelets accumulated on the PA12 particle surface in some areas, resulting in a non-homogeneous nanoclay distribution. The EC-based composite exhibits less accumulation and a much more homogeneous clay distribution.

### Testing the Mechanical Properties

**Fig. 5:**
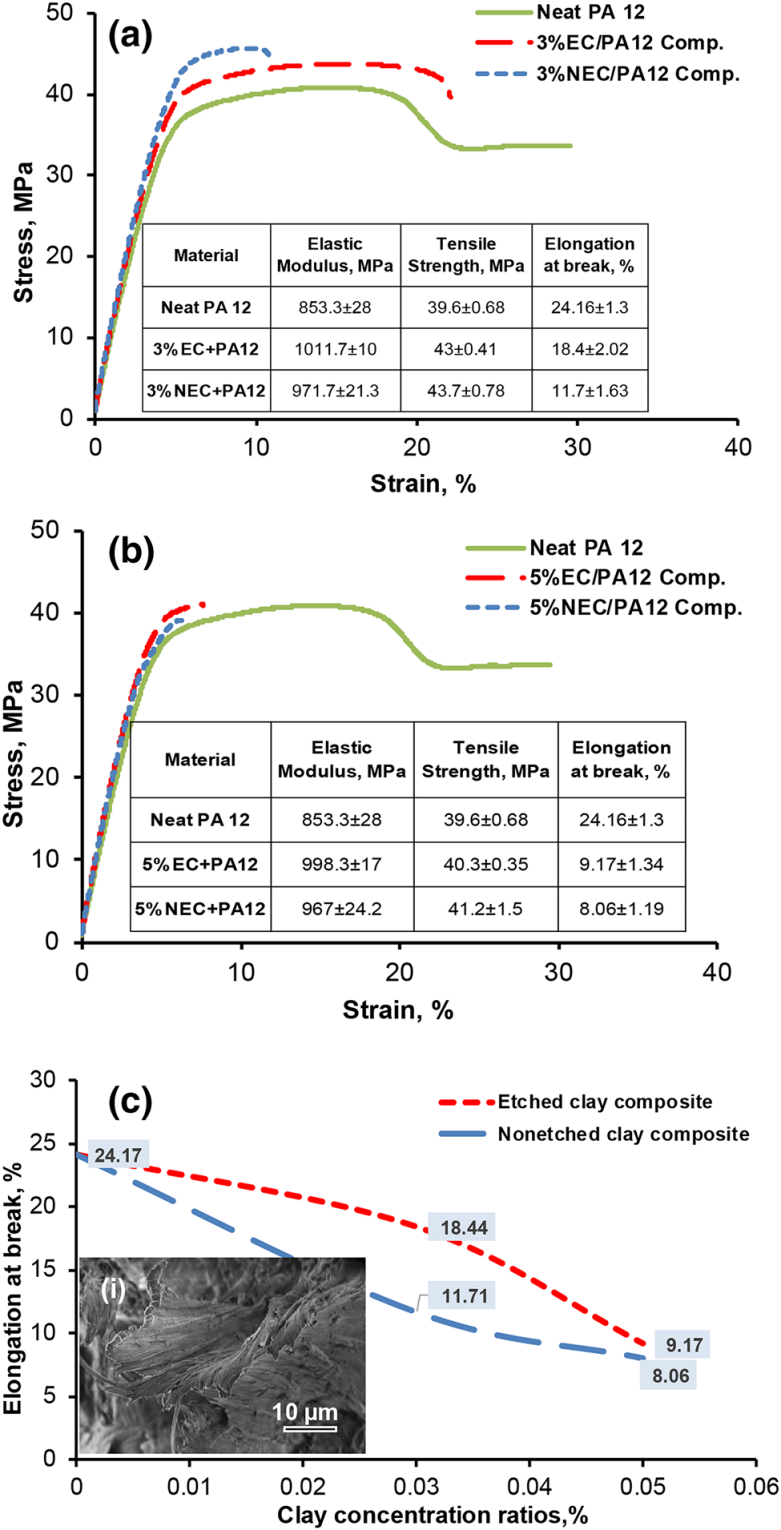
(a) Elastic modulus and (b) tensile strength of EC/PA12 and NEC/PA12 composites (each *curve* is almost the best in tensile strength). (c) The elongation at break values of EC/PA12 and NEC/PA12 composites, and the SEM image of 3%EC/PA12 composite showing features of ductile fracture (*inset i*)

Comparisons made between neat PA12, NEC/PA12, and EC/PA12 are summarized in Fig. 5a, and b and all data are accessible in Ref. 29. Compared to neat PA12, an improvement of the elastic modulus and strength was found for both NEC/PA12 and EC/PA12 at clay concentrations of 3% and 5%, whereas at the same time a reduction in the elongation at break was measured. Ultimately, a combination of tensile modulus, tensile strength and elongation must be considered.^30^ It was found in the current study that the best combination of these properties was obtained at 3% EC/PA12. As can be seen from the table in Fig. 5a, adding the EC at a concentration of 3% has increased the elastic modulus and tensile strength by ~19% and ~9%, respectively (compared with neat PA12), with a simultaneous reduction in the elongation at break by ~24%. Both exceed the performance of clay/PA12 laser sintered nanocomposites with the same clay loading reported in Ref. 5. The elongation of EC/PA12 composite that decreased by (~24%) is smaller than that obtained from the NEC/PA12, which is ~52% (see Fig. 5c). The SEM gave further evidence of the ductile fracture for EC/PA12 as shown in Fig. 5c (inset i). Incorporation of the rigid clay strengthens the matrix polymer but it also leads to a reduced ductility and brittle fracture, as expected.^14^ In addition, the poor interaction between the nonorganic clay and organic polymer is not enough to resist the axial force. Micro-voids will be presented as a result of the bad dispersion.^17^ The micro-voids may develop to initiate a micro-crack and the propagated cracks will lead to a brittle fracture. Hence, our results suggest that the EC may have a stronger interaction with PA12 than the NEC. A notable result from the tensile testing is the reduction in the variation of the elastic modulus results between different specimens, but only in the case of adding the EC to PA12, as shown in the tables of Fig. 5. This is attributed to a more homogeneous distribution and better dispersion of the EC within the PA12 powders and ultimately the composite (as evidenced by the SEM images of powders and fracture surfaces, respectively.).

The incorporation of clay at high concentration resulted in less strengthening^5^ and reduced ductility.^17^ Similarly, our results at 5% concentration showed that the strength and elastic modulus hardly improved. Moreover, the elongation at break was decreased dramatically by 62% (EC/PA12) compared with PA12.

Figure S1(a and b) (supporting information) from the SEM images at low magnifications show the difference between two fracture surfaces: (1) the NEC-based composite exhibits brittle fracture areas, and (2) the EC fracture surface (second fracture) shows a more ductile and uniform surface, presumably due to the avoidance of micron-sized agglomerates, which was the main aim of this work. Further optimization of the plasma treatment should focus on the nano-scale dispersion (e.g., exfoliation and intercalation), which will be investigated in future work.

## Conclusion

Hot-stage microscopy has been successfully used to determine suitable processing temperatures to fabricate nanoclay-Polyamide 12 composites, while DSC has been shown to be less suited for process optimization, as it did not clearly reveal the difference in melting behavior for composite powders.

The nanoclay/Polyamide 12 composites obtained with powders made in a dry mixing process of plasma-treated nano-clay with PA12 through downward heat sintering compare favorably to other mixing processes described in the literature, and are therefore encouraging for the use in laser sintering. Downward heat sintering was used to predict a suitable powder bed temperature, which was successfully applied to the laser sintering of the nanocomposite powders.

The current problem addressed is the avoidance of the micron-scale aggregates, which has been achieved using a plasma treatment technique. It has been demonstrated that large clay aggregates can be avoided through the use of plasma treatment leading to smaller variations in mechanical properties between different test specimens.

## Electronic supplementary material

Below is the link to the electronic supplementary material.

Supplementary material 1 (TIFF 20138 kb)

Supplementary material 2 (TIFF 20204 kb)

Supplementary material 3 (TIFF 50677 kb)
