# Supplementary figures and images for: Nanoclay/Polymer Composite Powders for Use in Laser Sintering Applications: Effects of Nanoclay Plasma Treatment
Source: JOM (1989). 2017 Jun 12;69(11):2278–85. doi: 10.1007/s11837-017-2408-5 (PMC6954011; doi:10.1007/s11837-017-2408-5)

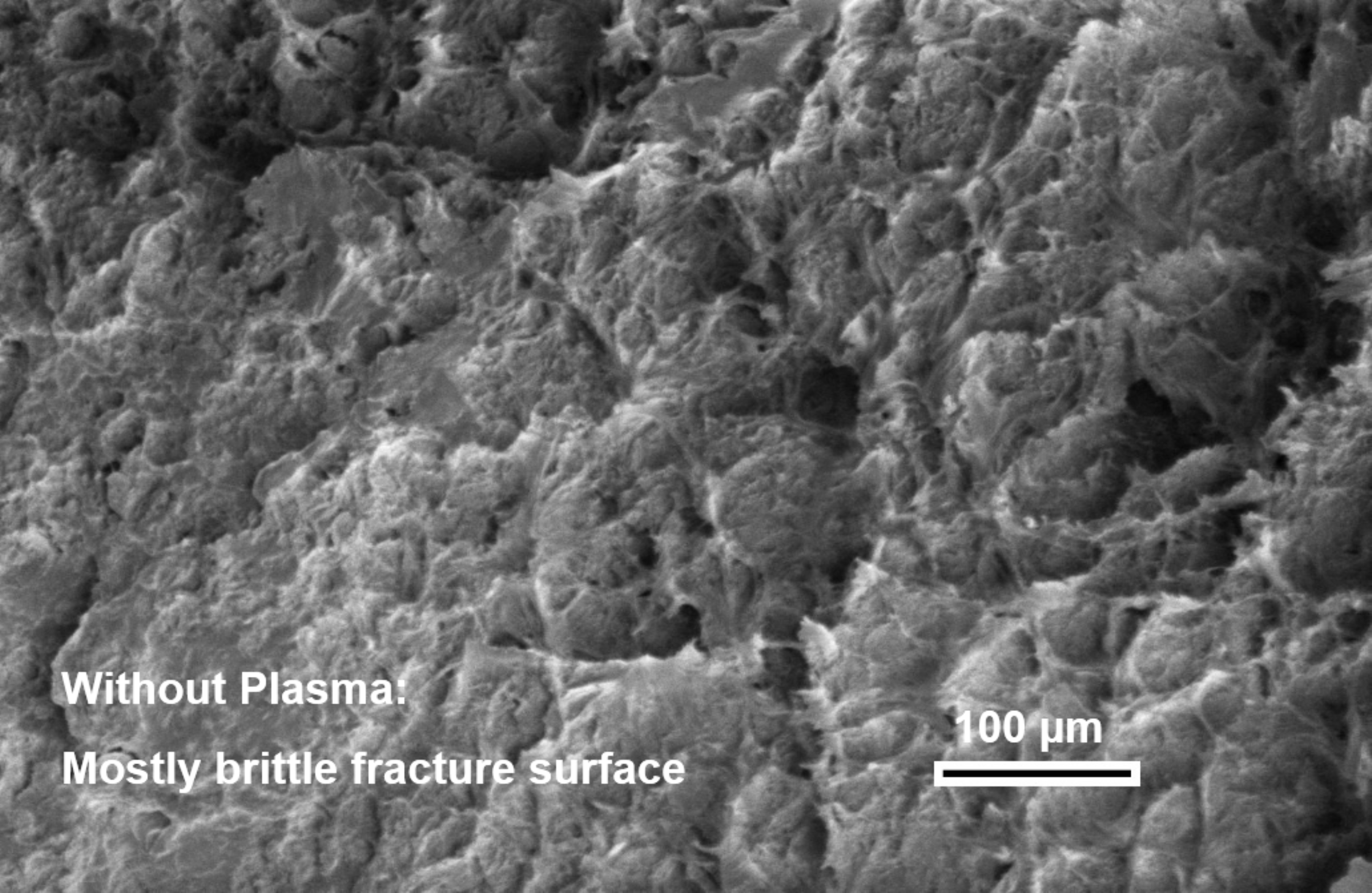

Supplement: Supplementary file 1 — Supplementary material 1 (TIFF 20138 kb) [file 11837_2017_2408_MOESM1_ESM.tif]

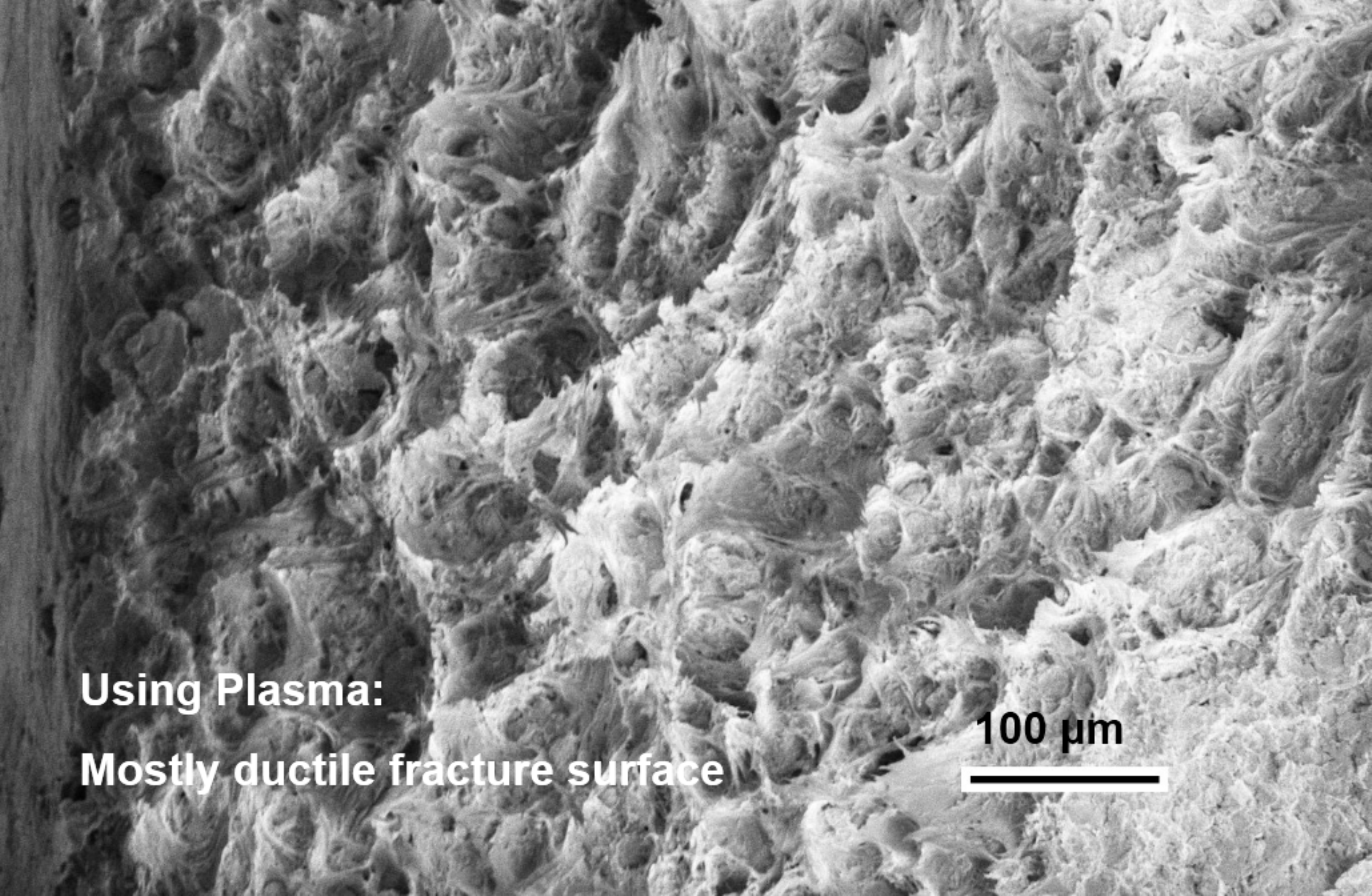

Supplement: Supplementary file 2 — Supplementary material 2 (TIFF 20204 kb) [file 11837_2017_2408_MOESM2_ESM.tif]

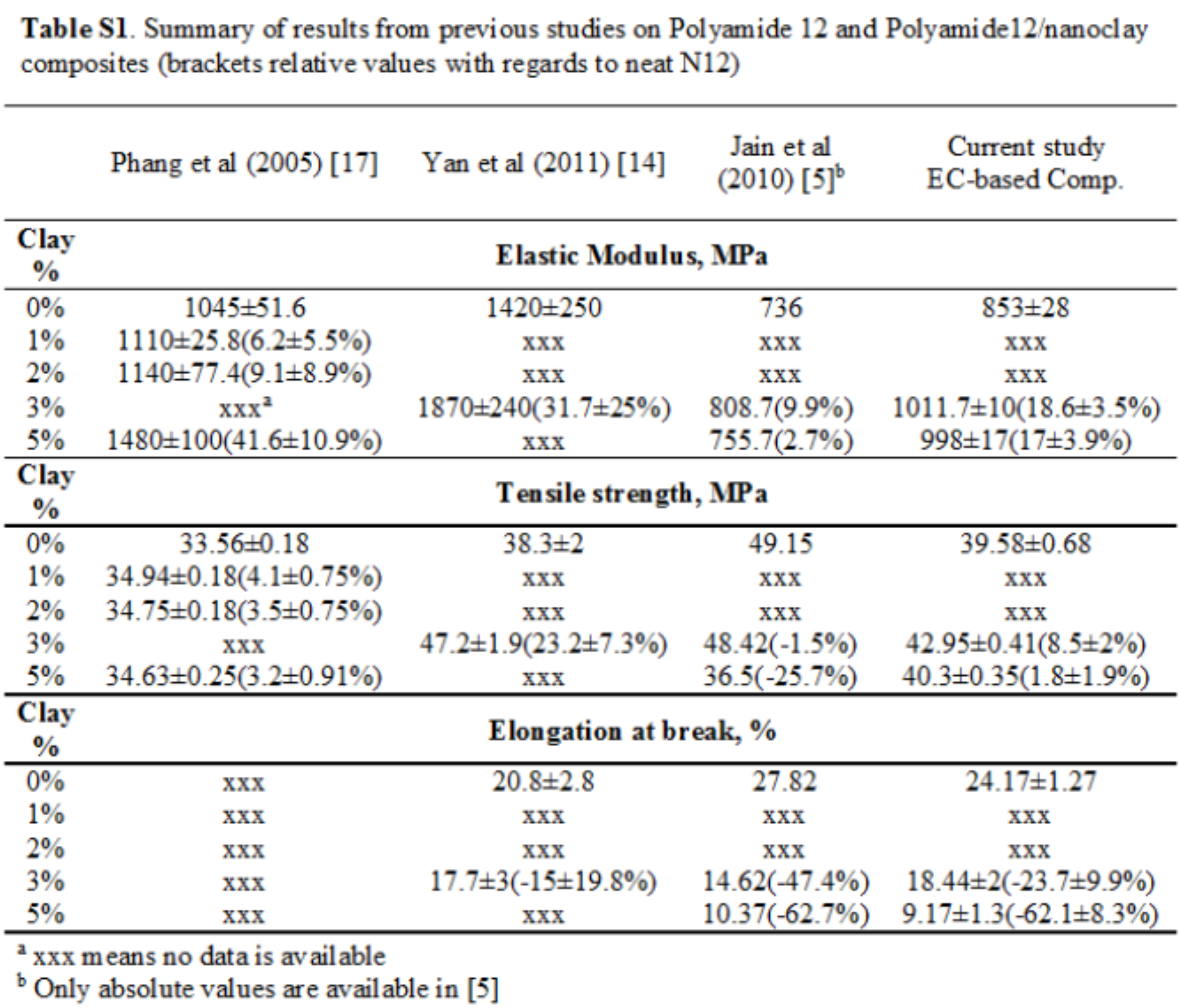

Supplement: Supplementary file 3 — Supplementary material 3 (TIFF 50677 kb) [file 11837_2017_2408_MOESM3_ESM.tif]
